# Exploring the causal relationship between glutamine metabolism and leukemia risk: a Mendelian randomization and LC-MS/MS analysis

**DOI:** 10.3389/fimmu.2024.1418738

**Published:** 2024-07-10

**Authors:** Na Li, Tianyi Wang, Huiying Zhang, Xiao Li, Haochen Bai, Ning Lu, Kaizhi Lu

**Affiliations:** ^1^ Mass Spectrometry Research Institute, Beijing Gobroad Hospital, Beijing, China; ^2^ Mass Spectrometry Research Institute, Beijing Gobroad Healthcare Group, Beijing, China; ^3^ Beijing Institute of Heart Lung and Blood Vessel Disease, Beijing Anzhen Hospital, Capital Medical University, Beijing, China; ^4^ Department of Laboratory Medicine, Beijing Gobroad Hospital, Beijing, China; ^5^ Mass Spectrometry Research Institute, Shanghai Liquan Hospital, Shanghai, China

**Keywords:** leukemia, glutamine, Mendelian randomization, LC-MS/MS, asparaginase treatment, therapeutic drug monitoring, personalized medicine

## Abstract

**Objective:**

This investigation sought to delineate the causal nexus between plasma glutamine concentrations and leukemia susceptibility utilizing bidirectional Mendelian Randomization (MR) analysis and to elucidate the metabolic ramifications of asparaginase therapy on glutamine dynamics in leukemia patients.

**Methods:**

A bidirectional two-sample MR framework was implemented, leveraging genetic variants as instrumental variables from extensive genome-wide association studies (GWAS) tailored to populations of European descent. Glutamine quantification was executed through a rigorously validated Liquid Chromatography-Mass Spectrometry/Mass Spectrometry (LC-MS/MS) protocol. Comparative analyses of glutamine levels were conducted across leukemia patients versus healthy controls, pre- and post-asparaginase administration. Statistical evaluations employed inverse variance weighted (IVW) models, MR-Egger regression, and sensitivity tests addressing pleiotropy and heterogeneity.

**Results:**

The MR findings underscored a significant inverse association between glutamine levels and leukemia risk (IVW p = 0.03558833), positing lower glutamine levels as a contributory factor to heightened leukemia susceptibility. Conversely, the analysis disclosed no substantive causal impact of leukemia on glutamine modulation (IVW p = 0.9694758). Notably, post-asparaginase treatment, a marked decrement in plasma glutamine concentrations was observed in patients (p = 0.0068), underlining the profound metabolic influence of the therapeutic regimen.

**Conclusion:**

This study corroborates the hypothesized inverse relationship between plasma glutamine levels and leukemia risk, enhancing our understanding of glutamine’s role in leukemia pathophysiology. The pronounced reduction in glutamine levels following asparaginase intervention highlights the critical need for meticulous metabolic monitoring to refine therapeutic efficacy and optimize patient management in clinical oncology. These insights pave the way for more tailored and efficacious treatment modalities in the realm of personalized medicine.

## 1 Introduction

Leukemia is a group of cancers that originate in the bone marrow, leading to the overproduction of abnormal white blood cells (1, 2). These cancerous cells can impede the bone marrow’s ability to produce red blood cells, platelets, and normal white blood cells, essential for carrying oxygen, clotting, and fighting infections, respectively (3). Characterized by its varied types, leukemia can be acute, with rapid progression, or chronic, with slow development over time. The disease’s etiology involves a combination of genetic and environmental factors, highlighting the importance of research for advanced diagnostic and therapeutic strategies (4, 5).

Glutamine, a non-essential amino acid, plays a pivotal role in various cellular processes, including energy production, biosynthesis, and regulation of signal transduction pathways (6, 7). In the context of cancer, glutamine metabolism is often reprogrammed to meet the increased demands of rapidly proliferating tumor cells, suggesting its potential involvement in cancer pathophysiology, including leukemia (8–10). This has spurred interest in exploring glutamine’s role and its metabolic pathways as therapeutic targets.

Investigating the causal relationship between specific biomarkers and diseases, such as the link between glutamine levels and leukemia, necessitates methodologies that circumvent the limitations of traditional observational studies, which are often susceptible to reverse causation and confounding factors. Mendelian randomization (MR) emerges as a robust epidemiological tool in this regard, leveraging genetic variants as instrumental variables (IVs) to infer causal relationships between exposures (e.g., glutamine levels) and outcomes (e.g., leukemia risk) (11, 12). MR relies on the principles that the genetic variants are strongly associated with the exposure, independent of confounders, and influence the outcome solely through the exposure, thus offering a genetic approach to causal inference that can complement and extend findings from genome-wide association studies (GWAS) (13–15).

The methodology for monitoring and analyzing glutamine levels, alongside the pharmacokinetics of therapeutic agents, employs advanced techniques such as High-Performance Liquid Chromatography-Tandem Mass Spectrometry (HPLC-MS/MS) (16). This analytical method provides a highly sensitive and specific measurement of glutamine concentrations in blood, facilitating the precise monitoring of metabolic changes in response to therapy, including Therapeutic Drug Monitoring (TDM) of treatments in leukemia patients (17, 18). TDM is instrumental in optimizing drug dosages to maximize efficacy while minimizing toxicity, thereby enhancing patient outcomes (19, 20).

Given the intricate relationship between glutamine metabolism and leukemia progression, and the potential impact of therapeutic interventions on glutamine dynamics, our study aims to explore the causal connection between glutamine levels and leukemia using a bidirectional MR approach. We developed and validated an LC-MS/MS method to quantify glutamine in blood, comparing concentrations between leukemia patients and healthy controls. Additionally, we investigate the effect of asparaginase treatment on glutamine levels in leukemia, aiming to understand the metabolic consequences of leukemia therapies. Our research seeks to uncover insights into glutamine’s role in leukemia, offering potential directions for therapeutic intervention.

## 2 Materials and methods

### 2.1 Mendelian randomization

This investigation adhered to the Strengthening the Reporting of Observational Studies in Epidemiology (STROBE) guidelines (21) and its extension for MR studies, STROBE-MR (22). We utilized a bidirectional two-sample MR approach to examine the potential causal links between glutamine levels and leukemia risk. This included an initial phase assessing the influence of altered glutamine levels on the likelihood of developing leukemia, and a subsequent phase investigating the reverse association.

#### 2.1.1 Data sources

Our study leveraged GWAS datasets as of September 2021, obtained from the UK Medical Research Council’s (MRC) University of Bristol Integrated Epidemiology Unit (IEU), focusing on individuals of European ancestry to minimize population stratification effects. The analysis incorporated two updated datasets: one on leukemia (Dataset: ieu-b-4914, including 1,260 cases and 372,016 controls, Author: Burrows, Consortium: UK Biobank, aligned with the HG19/GRCh37 genomic build) and another on glutamine levels (Dataset: ebi-a-GCST90026170, with a sample size of 291, Author: Panyard DJ, consisting of 6,839,564 SNPs). These datasets ensure genetic reference consistency and relevance to our research focus on leukemia and glutamine metabolism. The general information of the dataset is shown in **Table 1**.

**Table 1:** General information about the datasets.

Phenotype	Group ID	Database	Year	Number of SNPs	Population
leukemia	ieu-b-4914	IEU GWAS	2021	9,880,879	European
glutamine levels	ebi-a-GCST90026170	IEU GWAS	2021	6,839,564	European

#### 2.1.2 Selection of genetic IVs

IVs for glutamine levels and leukemia were identified through SNP filtering, applying a significance threshold (p<5×10^-8^) and independence criteria (R^2 < 0.001 within a 10,000 kb window) to minimize linkage disequilibrium. This step involved the use of the clumping function in the Two-Sample MR package in R, excluding SNPs associated with potential confounders or outcomes other than leukemia, based on PhenoScanner database queries. SNPs with palindromic alleles or indicative of weak instruments (F statistic < 10) were also excluded to prevent ambiguity and ensure robust instrument strength.

#### 2.1.3 Mendelian randomization analysis

The MR analysis was executed using R (version 4.1.0), incorporating both the Two-Sample MR and MR-PRESSO packages for thorough data examination. The analytical framework was structured to harmonize exposure and outcome datasets, facilitating comparability. A detailed flowchart of the MR analysis process was included for clarity and transparency.

#### 2.1.4 Sensitivity analysis

Sensitivity analyses, including MR-Egger regression and the MR-PRESSO test for horizontal pleiotropy, were conducted to evaluate the robustness of our findings and address potential biases. Cochran’s Q test assessed heterogeneity among IVs, and a “leave-one-out” approach was implemented to determine the impact of individual SNPs on the overall analysis. This comprehensive approach ensured the reliability and validity of our conclusions regarding the causal relationship between glutamine levels and leukemia risk.

#### 2.1.5 Statistical analysis

Multiple MR methods were employed, with the Inverse Variance Weighted (IVW) approach serving as the primary analysis tool, offering unbiased causal estimates in the absence of horizontal pleiotropy. Additional methods—Simple Mode, MR-Egger regression, Weighted Mode, and Weighted Median—were utilized to affirm the analysis’s robustness. Statistical significance was determined at a p-value < 0.05, with a Bonferroni adjustment for multiple testing to control for type I error.

### 2.2 LC-MS/MS

#### 2.2.1 Chemical reagents and reagents

L-Asparagine (#C2302017) and DL-Glutamine (#D2126337) standards were purchased from Shanghai Aladdin Biochemical Technology Co., Ltd., with purities of 99% and 100%, respectively. Isotope-labeled internal standards, L-Asparagine-^15^N_2_ (#23Z420-G1) and L-Glutamine-^13^C_5_ (#21J168-R7), were obtained from Shanghai Standard Biotechnology Co., Ltd., with purities of 98.5% and 98% respectively. All reagents were stored at 2–8°C until use. Methanol and formic acid were acquired from Thermo Fisher Scientific.

#### 2.2.2 Chromatographic and mass spectrometric conditions

Chromatographic separation was conducted on HPLC system equipped with a ChromCore HILIC-Amide column (3µm, 2.1x100mm). The column temperature was maintained at 40°C. The mobile phase consisted of 0.1% formic acid in water (A) and 0.1% formic acid in acetonitrile (B), with a flow rate of 0.4 mL/min. The gradient program starts at 90% B, drops to 50% B in 2.5 minutes, rises to 90% B at 3.1 minutes, and is held until 4 minutes.

The mass spectrometer used was the YS EXT 9900 MD Triple Quadrupole Mass Spectrometer from Shandong Yingsheng Biotechnology Co., Ltd. Ionization was performed in the positive ion mode using an Electrospray Ionization (ESI) source. Detection was performed using Selected Reaction Monitoring (SRM). Key operational settings included an ion spray voltage of 3500 V, sheath gas flow at 45 arbitrary units, auxiliary gas flow at 15 arbitrary units, and sweep gas flow at 5 arbitrary units. The ion transfer tube temperature was set at 350°C, and the vaporizer temperature at 450°C. Quadrupole resolutions for Q1 and Q3 were maintained at a Full Width at Half Maximum (FWHM) of 0.7.

Multiple Reaction Monitoring (MRM) mode was used to monitor transitions from m/z 133.1 to 74.1 and 87.1 (Asparagine), m/z 135.1 to 75.1 and 89.1 (Asparagine-^15^N_2_), m/z 147.1 to 84.1 and 130.1 (Glutamine), and m/z 152.1 to 88.1 and 135.1 (Glutamine-^13^C_5_). The collision energies were set at 16V and 10V for Asparagine and Asparagine-^15^N_2_, and 25V and 15V for Glutamine and Glutamine-^13^C_5_, respectively.

#### 2.2.3 Method validation

Method validation was conducted according to international guidelines covering specificity, linearity, accuracy, precision, recovery, matrix effects, and stability. Calibration curves were linear across the range using a 1/x weighting factor. Precision and accuracy for intra-day and inter-day were assessed using six replicates of quality control (QC) samples at three different times.

#### 2.2.4 Sample pre-treatment

50 μL of clinical plasma samples were mixed with 150 μL of internal standard solution, vortexed for 1 minute, and then centrifuged at 14,000 rpm for 10 minutes at 4°C. The supernatant (150 μL) was transferred to sample vials. Prepared samples were then analyzed using LC-MS/MS.

#### 2.2.5 External quality control assessment

To enhance the validation of the HPLC-MS/MS methodology for quantifying glutamine, we implemented external QC measures by incorporating amino acid QC samples provided by PRIZE.BIO. These QC samples, specifically engineered for bioanalytical method validation, encompassed two concentration levels: a Low-Quality Control (LQC), where glutamine and asparagine concentrations were 30.6 μmol/L and 9.9 μmol/L, respectively; and a High-Quality Control (HQC), with concentrations of 755.2 μmol/L for glutamine and 73.5 μmol/L for asparagine.

### 2.3 Quantification of plasma glutamine levels

#### 2.3.1 Study participants and sampling

Patients with leukemia undergoing chemotherapy protocols were recruited for this study. As part of their treatment, asparaginase was administered at doses ranging from 40 to 200 U/kg through intramuscular injections, either daily or every other day, for a total of 3 to 7 doses per week. A single therapy cycle lasted 3 to 4 weeks. Plasma samples were strategically collected to align with specific pharmacodynamic parameters of asparaginase treatment; samples were obtained immediately before the first dose of asparaginase, and subsequent samples were taken 24 hours post-administration, once asparaginase levels decreased to below 0.5μmol/L, the threshold for optimal therapeutic efficacy, to accurately determine plasma glutamine concentrations. These collection points were selected to directly link asparaginase activity to changes in glutamine levels. Plasma glutamine concentrations from healthy volunteers were also measured to serve as baseline reference values. All study procedures adhered to ethical standards as per the guidelines of the institutional and national research committee, in accordance with the 1964 Helsinki declaration and its later amendments. Informed consent was obtained from all participants.

#### 2.3.2 Plasma preparation

Blood samples were drawn into tubes containing EDTA, mixed gently, and centrifuged at 1500×g for 15 minutes at 4°C to isolate the plasma. The separated plasma was aliquoted and stored at -80°C to prevent analyte degradation, including measures to avoid repeated freeze-thaw cycles.

#### 2.3.3 LC-MS/MS analysis and method validation

Identical to Section 2.2.

#### 2.3.4 Statistical analysis

Data on glutamine concentrations were statistically evaluated and expressed as mean ± SD. Differences in plasma glutamine levels pre- and post-asparaginase treatment in leukemia patients, and between leukemia patients and healthy controls, were determined using suitable statistical tests (e.g., paired t-test, ANOVA), with an alpha level of significance established beforehand.

## 3 Results

### 3.1 Impact of glutamine levels on leukemia risk

#### 3.1.1 Selection of IVs

Adhering to our criteria, we selected IVs that demonstrated no linkage disequilibrium (LD; r^2 < 0.001) and were within a physical distance threshold of 10,000 kb, achieving genome-wide significance (p<5×10^-8). After applying the Pheno-Scanner tool and setting a minor allele frequency (MAF) threshold (>0.01) for the removal of palindromic SNPs, our analysis incorporated 9 SNPs. The F-statistics of these SNPs were all greater than 10, eliminating the concern of weak tools. Detailed SNP information related to glutamine levels is available in **Supplementary Table S1**.

#### 3.1.2 Two-Sample Mendelian Randomization Analysis

**Figure 1:**
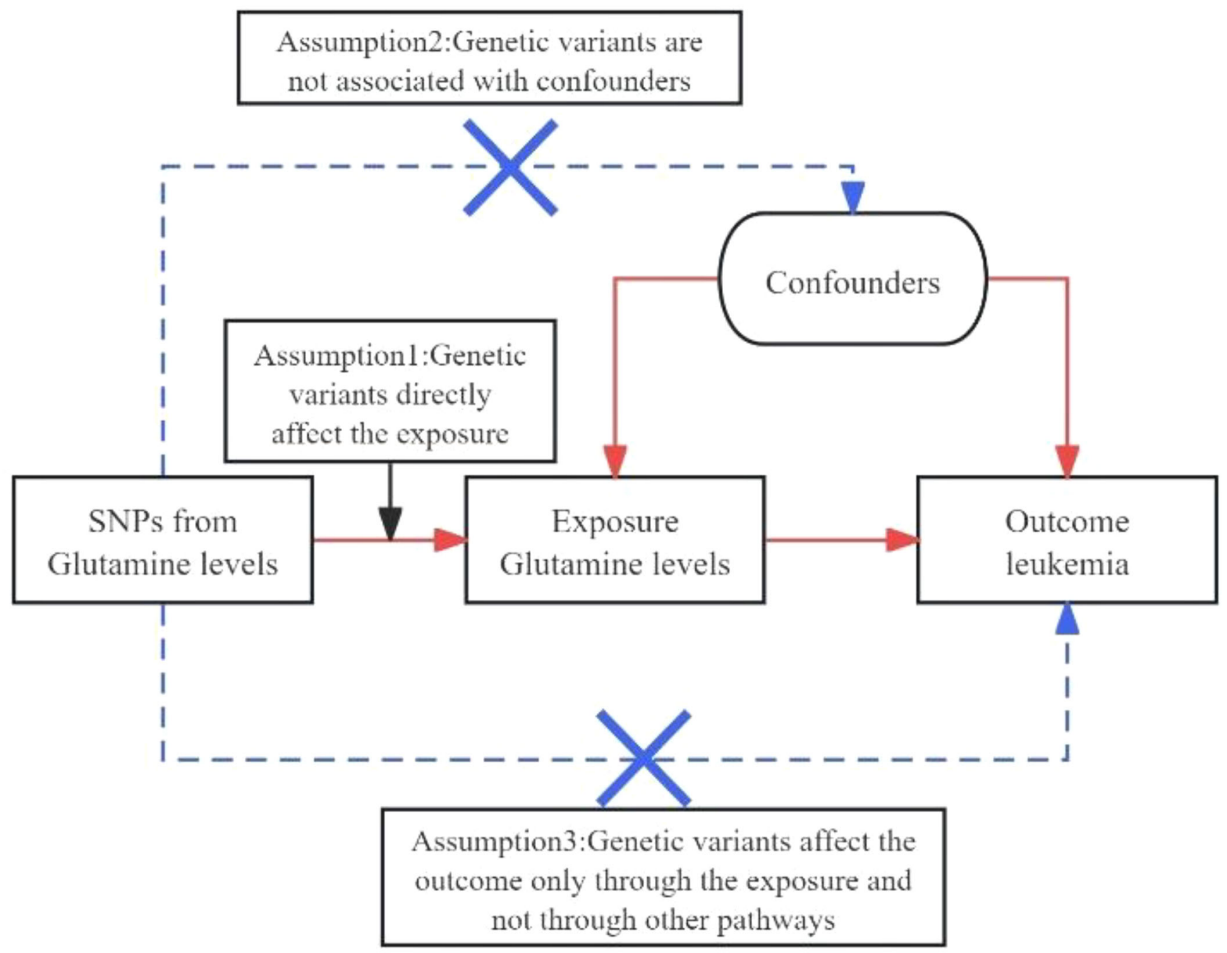
Diagrams A and B for bidirectional MR analysis. SNPs meet three basic assumptions. MR, Mendelian randomization; SNPs, single nucleotide polymorphisms;.

**Figure 2:**
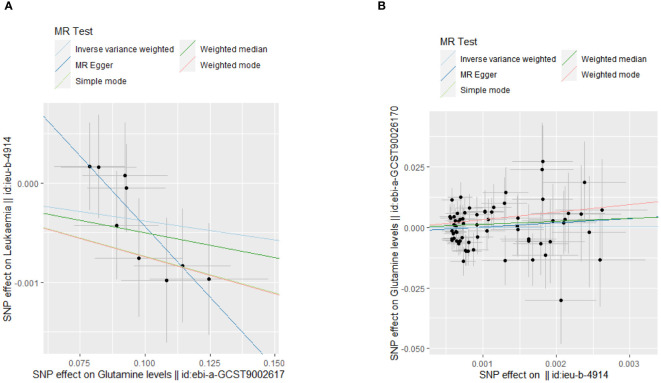
MR analysis scatter plot. **(A)** The causal effect of glutamine levels on leukemia; **(B)**The causal effect of leukemia on glutamine levels.

**Figure 3:**
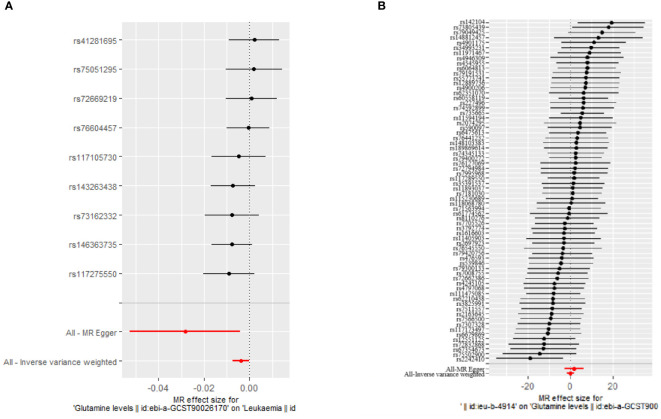
Forest plot showing specific SNPs and combined MR estimates. **(A)** The causal effect of glutamine levels on leukemia; **(B)** The causal effect of leukemia on glutamine levels.

Our MR analyses employed five distinct methods to examine the causal effect of glutamine levels on leukemia risk. The analyses identified a statistically significant inverse relationship, indicating that reduced glutamine levels are associated with the risk of developing leukemia. The IVW method presented a causal estimate of -0.003821051 (p = 0.03558833), suggesting reduced glutamine levels are associated with an increased risk of leukemia. This finding was consistent across various MR methods, including MR Egger and Weighted Median, thereby confirming the robustness of our results. Tests for horizontal pleiotropy did not show significant pleiotropy (Egger’s intercept p = 0.08545201), implying that the associations observed are unlikely to be influenced by unmeasured pleiotropic effects. The heterogeneity analysis also endorsed the consistency of our findings (IVW Q_pval = 0.6948101). The detailed analysis process is shown in **Figure 1** and the results of the analysis are shown in **Tables 2** and **3**. And the MR estimation of the causal relationship between glutamine level and leukemia is shown in **Figure 2**. Forest plots showing specific SNP and combined MR estimates are shown in **Figure 3**.

**Table 2:** Different MR estimation methods for assessing the causal effect of glutamine levels and leukemia.

	No. SNPs	IVW^a^			weighted median^a^			MR-Egger^a^			weighted mode^a^		
		95%CI	Beta	*p*	95%CI	Beta	*p*	95%CI	Beta	*p*	95%CI	Beta	*p*
G→L	9	-0.01,1,00	-0.003821051	0.03558833	-0.01,1.00	-0.005010792	0.04260482	-0.05,1.00	-0.028375670	0.05599062	0.98,1.00	-0.007429780	0.09796156
L→G	68	0.18,6.42	0.03559825	0.9694758	0.22,6.42	1.23491100	0.3753537	0.05,3.92	1.72423007	0.4678621	0.04,2.28	3.12010762	0.3391333

G→L: the causal effect of glutamine levels on leukemia risk; L→G: the causal effect of leukemia on glutamine levels risk. a: no MR-PRESSO outliers were exposed.

**Table 3:** Heterogeneity and horizontal pleiotropy check between glutamine levels with leukemia.

Exposure	Outcome	Horizontal pleiotropy test (MR-Egger)			Heterogeneity test (IVW)		Heterogeneity test (MR-Egger)	
		Intercept	SE	P	Q	P	Q	P
Glutamine levels	Leukemia	0.002402388	0.001200398	0.08545201	5.574164	0.6948101	1.568857	0.9798296
Leukemia	Glutamine levels	-0.001593354	0.002046821	0.4390839	68.14460	0.4380670	67.52462	0.4247919

### 3.2 Effect of leukemia on glutamine levels

#### 3.2.1 Selection of IVs

Applying the same stringent selection criteria for IVs as detailed earlier, 68 SNPs were identified post-removal of palindromic sequences. These SNPs were rigorously evaluated, displaying an F-statistic well above the threshold, indicating it as a strong instrument for studying the effect of leukemia on glutamine levels.

#### 3.2.2 Two-sample Mendelian randomization analysis

The MR analysis, aimed at understanding the influence of leukemia on glutamine levels, did not uncover any significant causal relationships across the employed methods (IVW, MR Egger, Weighted Median). The IVW method, for instance, yielded a causal estimate of 0.03559825 (p = 0.9694758), suggesting no significant effect of leukemia on altering glutamine levels. Consistency in findings was observed across additional MR methods employed in our analysis. The lack of observed heterogeneity (IVW Q_pval = 0.4380670) and horizontal pleiotropy (p > 0.05) lends further credibility to the null findings. Detailed results are documented in **Supplementary Table S2** and visually represented in **Tables 2** and **3**.

### 3.3 LC-MS

#### 3.3.1 Specificity

**Figure 4:**
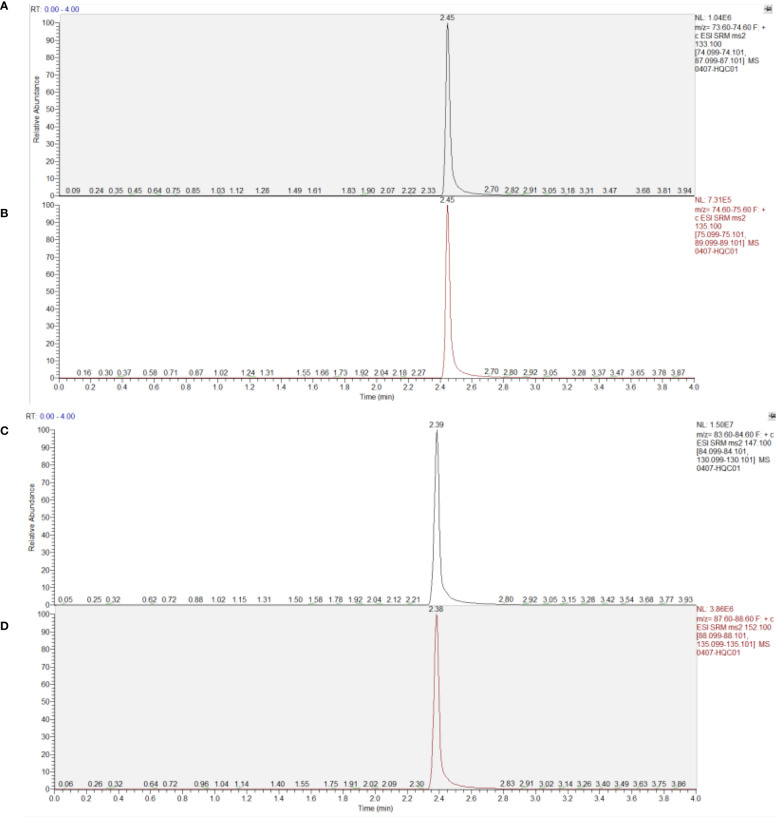
Chromatogram of substances in plasma samples. **(A)** Asparagine; **(B)** Asparagine-^15^N_2_; **(C)** Glutamine; **(D)** Glutamine-^13^C_5_.

As depicted in **Figure 4**, under the aforementioned conditions, the retention times for Asparagine, Asparagine-^15^N_2_, Glutamine, and Glutamine-^13^C_5_ were 2.46, 2.46, 2.39, and 2.38 minutes respectively. The high-resolution mass spectrum can be seen in **Figure 4**.

#### 3.3.2 Standard curve and lower limit of quantitation

L-asparagine demonstrated a good linear relationship within the range of 0.4 to 100.0 μmol/L. The standard curve equation was defined as: y=8.778*10^-2^x-2.479*10^-3^(R²=0.9993) with a lower limit of quantitation of 0.4 μmol/L. For Glutamine, the linear relationship was also strong within the range of 4 to 1000.0 μmol/L, with the standard curve equation: y=2.225*10^-2^x-9.219×10^-3^(R²=0.9995) and the lower limit of quantitation was set at 4 μmol/L.

#### 3.3.3 Precision and accuracy

For quality control samples at low, medium, and high concentrations, the intra-day and inter-day standard deviations (SD) for both asparagine and glutamine were within 5%. The specific data are presented in **Tables 4** and **5**.

**Table 4:** Precision and accuracy of glutamine quantification by mass spectrometry in method validation.

	First batch (N=6)				Second batch (N=6)				Third batch (N=6)				Summary (N=18)			
	Mean	SD	%CV	%DEV	Mean	SD	%CV	%DEV	Mean	SD	%CV	%DEV	Mean	SD	%CV	%DEV
QCL(12μM)	11.91	0.08	0.67	-0.72	11.72	0.11	0.91	-2.35	11.71	0.16	1.4	-2.44	11.78	0.15	1.27	-1.84
QCM(150μM)	148.4	0.84	0.57	-1.1	145.1	1.08	0.74	-3.28	145	0.94	0.65	-3.33	146.1	1.84	1.26	-2.57
QCH(750μM)	765.1	3.98	0.52	2.02	760.2	4.71	0.62	1.36	761.4	3.77	0.5	1.53	762.3	4.47	0.59	1.64

Mean: Intra-batch average; SD: Within-batch standard deviation; %CV: Within-batch precision; %DEV: Within-batch accuracy deviation.

**Table 5:** Precision and accuracy of asparagine quantification by mass spectrometry in method validation.

	First batch (N=6)				Second batch (N=6)				Third batch (N=6)				Summary (N=18)			
	Mean	SD	%CV	%DEV	Mean	SD	%CV	%DEV	Mean	SD	%CV	%DEV	Mean	SD	%CV	%DEV
QCL(1.2μM)	1.21	0.03	2.33	1.04	1.22	0.01	0.45	1.5	1.2	0.03	2.49	0.14	1.21	0.02	1.95	0.89
QCM(15μM)	14.6	0.08	0.57	-2.65	14.5	0.21	1.45	-3.44	14.7	0.13	0.91	-1.98	14.6	0.17	1.16	-2.69
QCH(75μM)	75.3	0.8	1.06	0.44	74.3	0.89	1.19	-0.88	75.1	0.57	0.75	0.13	74.9	0.84	1.12	-0.11

#### 3.3.4 Stability

The recovery rates for simulated plasma samples at low, medium, and high concentrations remained above 90% after 12 hours in the autosampler, three freeze-thaw cycles, and 30 days of storage at -20°C. The relative standard deviations (RSD) were below 10%, indicating the stability met the requirements for determination.

#### 3.3.5 Outcomes of external quality control testing

LQC Results: For glutamine at Level-1, with a target value of 30.6 μmol/L, the measured concentration was 29.8 μmol/L, resulting in a deviation of -2.61%. This level of accuracy demonstrates the assay’s precision within its lower sensitivity range. For asparagine at the same level, a target value of 9.9 μmol/L yielded a measured concentration of 10.4 μmol/L, translating to a deviation of 5.05%, indicating satisfactory recovery and precision.

HQC Results: At the higher concentration Level-2, glutamine’s target of 755.2 μmol/L compared to the measured value of 723.6 μmol/L shows a deviation of -4.18%, reflecting consistent performance even at elevated levels. Asparagine, targeted at 73.5 μmol/L, had a measured concentration of 70.4 μmol/L with a deviation of -4.22%, confirming the method’s robustness across its operational range. Detailed results are in **Table 6**.

**Table 6:** QC acceptance and LC-MS measured values for glutamine and asparagine.

	Concentration level	QC acceptance range			LC-MS measured value	
		Target value	Low value	High value	Measured value	Deviation (%)
Glutamine (μmol/L)	Level-1	30.6	24.6	36.8	29.8	-2.61
	Level-2	755.2	604.2	908.8	723.6	-4.18
Asparagine (μmol/L)	Level-1	9.9	7.9	11.8	10.4	5.05
	Level-2	73.5	59.8	90	70.4	-4.22

### 3.4 Plasma glutamine concentrations

Our research ventured into analyzing plasma glutamine levels across distinct groups, including healthy subjects, leukemia patients before chemotherapy, and leukemia patients after treatment with asparaginase. Healthy individuals showcased an average plasma glutamine concentration of 589.18 ± 34.14 μmol/L. Contrarily, before chemotherapy, leukemia patients displayed a mean glutamine level of 609.95 ± 205.83 μmol/L. Despite this slight increase, statistical analysis indicated that this elevation was not significantly different from the levels seen in healthy controls (p = 0.5342), suggesting that leukemia, per se, does not lead to a significant change in glutamine levels. This finding aligns with our MR analysis, which did not demonstrate a significant causal effect of leukemia on altering plasma glutamine levels, thereby suggesting a complex relationship between leukemia and glutamine metabolism that is not directly causal.

**Figure 5:**
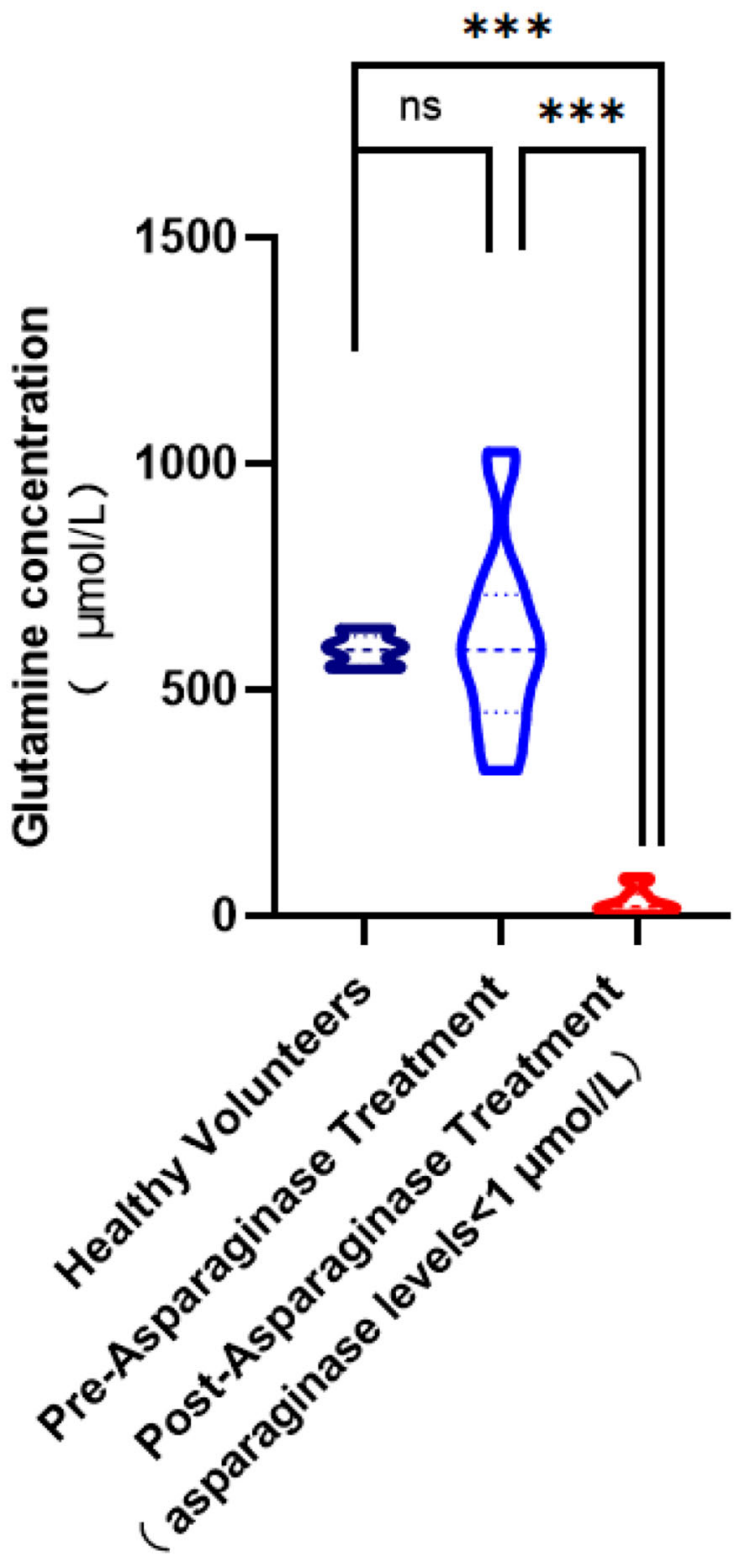
Comparison of glutamine concentration levels in plasma. (ns,no significance; ***, p< 0.001).

The primary effect of asparaginase is to deplete asparagine rather than glutamine. The administration of asparaginase was effective *in vivo*, reducing asparagine concentration to 1 μmol/L (23). We observed a significant reduction in plasma glutamine concentration to 33.31 ± 28.28μmol/L. This large decrease (p<0.0001) highlights the wide-ranging metabolic impact of asparaginase, which affects not only asparagine but also glutamine levels. The data statistics are shown in **Figure 5**.

## 4 Discussion

In this comprehensive study on the role of glutamate metabolism in leukemia, we integrated bidirectional MR analysis with precise LC-MS/MS quantitation techniques to unveil the complex interplay between glutamate levels and leukemia risk. Our bidirectional MR study examined the causal relationship between glutamate levels and the risk of leukemia, utilizing genetic variants as proxies for effect alleles selected from publicly available large-scale GWAS databases (24). To our knowledge, this is the first application of the bidirectional two-sample MR analysis method to this topic. Our findings indicate a statistically significant inverse relationship between glutamine levels and leukemia risk, suggesting that reduced glutamine levels may increase risk of leukemia. This relationship is not influenced by specific genetic susceptibilities to different glutamine levels, indicating a general protective effect against leukemia. Because observational epidemiologic studies can be subject to bias and confounders (25), the relationship between glutamine levels and leukemia risk is questionable.

In our analysis, significant findings were observed only with the IVW and Weighted Median methods, while other MR statistical approaches did not yield positive results. However, our conclusions remain robust as IVW and Weighted Median are among the most commonly utilized, principal, and persuasive methods in MR analysis. These methods have been validated as providing reliable estimates of causal effects, particularly in settings where the genetic instruments are valid and the underlying assumptions of MR are met (26). Additionally, despite the modest Beta values associated with these two methods, the p-values were less than 0.05, signifying statistical significance. This finding aligns with the discourse presented in “Principles of Biostatistics” by Marcello Pagano and Kimberlee Gauvreau, which illustrates that even small effect sizes, if statistically significant, are indicative of genuine effects and not merely due to random variation (27). The ability of small effect sizes to substantially improve health outcomes underscores the potential clinical significance of our results, suggesting that even minor modulations in glutamine levels could be integral to leukemia management and prevention strategies.

In our study, we leveraged strong SNPs from the largest GWAS on glutamine levels and leukemia, employing rigorous methodologies to control for pleiotropy and test for heterogeneity. Furthermore, by adhering to the principles of segregation and independent assortment in our MR approach, we minimized potential confounders and reverse causation risks (28). Thus, our MR findings are both accurate and stable, providing solid support for the evidence presented. Our analysis aligns with recent observational studies (29), confirming a causal relationship between high glutamine levels and reduced leukemia risk, although it does not support a causal link with other specific leukemia risk factors. Our findings are consistent with and expand upon existing research in the field. Recent foundational research has discovered that directly supplementing glutamine in tumor tissues can enhance CD8+ T cell-mediated antitumor immunity and overcome resistance to checkpoint blockade and T cell-mediated immunotherapies (30, 31). This underscores the critical role of glutamine in tumor cell metabolism, supporting energy synthesis and playing a significant role in inhibiting tumor growth (31–33). Moreover, the binding of glutamine transferase with targeted inhibitors has been shown to effectively kill tumor cells (10, 34). However, while the therapeutic use of glutamine in cancer patients is beneficial, careful management is required to avoid adverse effects such as liver dysfunction (7, 35). Our study, through MR, suggests that naturally elevated glutamine levels have a protective effect against the development of leukemia, complementing these insights and hinting at the complex interplay between metabolism and immune response in cancer. This is consistent with the concept that regulating glutamine levels can bring therapeutic benefits and suggests the potential for using natural metabolic variability for cancer prevention and treatment. As research continues to unfold the multifaceted role of glutamine in cancer, it becomes increasingly clear that understanding and manipulating this amino acid’s metabolism may be key to developing more effective cancer treatments and immunotherapies.

Furthermore, recent research by Guo et al. (2023) demonstrates that glutamine acts as a crucial metabolic checkpoint in the tumor microenvironment (TME), significantly influencing anti-tumor immunity through its effects on conventional dendritic cells type 1 (cDC1s) (36)​​. The study reveals that glutamine supplementation within the tumor milieu augments cDC1-mediated CD8+ T cell immunity, thereby inhibiting tumor growth and overcoming resistance to immune checkpoint blockade therapies​​. This finding suggests that adequate glutamine levels are essential for the optimal functioning of cDC1s, which are pivotal in priming and activating cytotoxic T cells against tumors. Mechanistically, tumor cells and cDC1s compete for glutamine uptake via the transporter SLC38A2, which modulates anti-tumor immunity. The deprivation of glutamine impairs cDC1 function, reducing their ability to present antigens and activate T cells​​. This competitive uptake underscores the significance of glutamine availability in maintaining immune surveillance and effective anti-tumor responses. In the context of leukemia, the depletion of glutamine might similarly disrupt the function of immune cells, particularly cDC1s, leading to an impaired immune response and increased leukemia risk. The TME in leukemia could create a nutrient-depleted environment, reducing glutamine availability and consequently hampering the immune system’s ability to target and eliminate leukemic cells. Furthermore, the FLCN-TFEB signaling axis plays a critical role in glutamine-mediated regulation of cDC1 function. Loss of FLCN in DCs impairs their ability to utilize glutamine effectively, leading to dysfunctional antigen presentation and T cell priming (36)​​. This pathway highlights the intricate link between nutrient sensing, metabolic regulation, and immune function, suggesting that targeting glutamine metabolism could enhance DC-mediated anti-leukemic responses.

Additionally, our study’s methodological advancements, particularly the development of a sensitive and specific LC-MS/MS method for monitoring plasma glutamine levels, mark a significant progress in the field of personalized medicine. This innovation is not merely a technical improvement but represents a pivotal shift towards more nuanced approaches in patient care, especially in oncology. Through precise personalized treatment plans, patient outcomes can be significantly improved while minimizing potential toxicity (37). The importance of accurately measuring metabolic changes in glutamine and asparagine levels in patients undergoing asparaginase treatment cannot be underestimated. These non-essential amino acids play a key role in cancer metabolism, with their availability affecting tumor growth and response to therapy (38, 39). Thus, in treatments using asparaginase to regulate asparagine levels, its reduction and indirect impact on glutamine levels can profoundly affect tumor dynamics. However, the effectiveness and safety of these treatments highly depend on the precise modulation of these amino acid’s levels, highlighting the necessity of accurate monitoring techniques like LC-MS/MS (40, 41). Therefore, we are optimistic that by developing and applying sensitive and specific methods to monitor key metabolites like glutamine and asparagine, we are laying the groundwork for the advancement of personalized oncology. Simultaneously, by enabling precise TDM, we promote a deeper understanding of metabolic and therapeutic interactions, potentially improving patient outcomes and providing insights that may lead to breakthroughs in cancer treatment paradigms (42, 43).

The phenomenon of concurrent decreases in asparagine and glutamine levels following asparaginase treatment, an interesting observation in our therapy studies, highlights the complex interplay of amino acid metabolism in cancer treatment. Asparaginase, a chemotherapy drug often used in some leukemia treatments, primarily targets asparagine, depleting its levels in the blood and corresponding tumor microenvironment (44, 45). We believe the concurrent reduction in asparagine and glutamine levels under asparaginase treatment may be attributed to several interconnected mechanisms: 1) Metabolic compensation: As asparagine is depleted, tumor cells may undergo metabolic reprogramming to alleviate the impact of asparagine loss (46). Given the structural similarity and shared metabolic pathways between asparagine and glutamine, cells might increase the utilization of glutamine, attempting to compensate for the loss of asparagine, leading to reduced glutamine levels (47); 2) Enhanced glutamine degradation: Asparaginase treatment may also indirectly enhance the breakdown of glutamine (48). Glutamine serves as the nitrogen donor for the synthesis of asparagine through asparagine synthetase. With the depletion of asparagine, there might be an increase in asparagine synthetase activity, attempting to synthesize asparagine from available glutamine, thus lowering glutamine levels (49); 3) Changes in amino acid transport: Asparaginase treatment may affect the expression or activity of amino acid transport proteins, particularly those involved in glutamine transport (50). Altered transporter activity might lead to increased efflux or reduced influx of glutamine in tumor cells, resulting in decreased intracellular and consequently plasma glutamine levels (51); 4) Immune system modulation: Asparaginase not only affects tumor cell metabolism but also impacts the immune system, which may be a significant consumer of glutamine during activation (52). The regulation of immune responses by asparaginase treatment may alter glutamine utilization patterns, thereby affecting its systemic levels (53). Understanding the reasons behind the dual decline in asparagine and glutamine levels caused by asparaginase treatment is crucial for optimizing cancer treatment regimens involving asparaginase. It not only aids in tailoring treatments based on individual metabolic characteristics but also helps in predicting and managing potential side effects due to altered amino acid levels. Future research focusing on how asparaginase affects glutamine metabolism will provide deeper insights into cancer cell metabolic vulnerabilities, paving the way for developing new treatment strategies based on these metabolic dependencies.

Despite the significant insights our study provides into the role of glutamine metabolism in leukemia and personalized treatment strategies, it is important to recognize its limitations. First, the method relying on LC-MS/MS for monitoring asparagine and glutamine levels, although innovative, may not fully capture the dynamics and complexity of cancer metabolism. Metabolic pathways are highly interconnected, and the impact of altering a single amino acid level may extend beyond our current understanding. Second, our study results are primarily based on European population data, and due to genetic variability, may not apply to different ethnic groups. This limitation emphasizes the need for further research across diverse populations to ensure the generalizability of our findings. Third, the bidirectional MR method, while powerful, inherently depends on the quality and depth of genetic variations used as IVs. Unidentified confounders or pleiotropy may affect the observed relationship between glutamine levels and leukemia risk. Additionally, our focus on glutamine and asparagine metabolism, although based on strong scientific reasoning, does not encompass all metabolic changes in cancer cells. Other metabolites and pathways may also play crucial roles in cancer progression and treatment response, requiring broader metabolic profiling methods. Finally, translating these biochemical and genetic insights into effective clinical treatments poses significant challenges. Developing targeted interventions that can precisely modulate glutamine and asparagine levels without adverse effects requires a deep understanding of their metabolic context in cancer, an understanding that is still evolving.

## 5 Conclusion

Our study underscores the crucial role of glutamine metabolism in the context of leukemia and its treatment, particularly highlighting the significant impact of asparaginase therapy on plasma glutamine levels. The findings advocate for the incorporation of glutamine monitoring alongside asparagine in patients undergoing asparaginase treatment, aiming to optimize therapeutic outcomes through precise metabolic control. This approach not only has the potential to enhance the efficacy of leukemia treatments but also minimizes adverse effects, contributing to the advancement of personalized medicine in oncology. Through a better understanding of the metabolic interplay involved in leukemia therapy, our study paves the way for more effective and tailored treatment strategies.

## Data availability statement

The original contributions presented in the study are included in the article/**Supplementary Material**. Further inquiries can be directed to the corresponding author.

## Ethics statement

The studies involving humans were approved by the Institutional Review Board of Beijing Gobroad Hospital, Beijing, China. The studies were conducted in accordance with the local legislation and institutional requirements. The participants provided their written informed consent to participate in this study.

## Author contributions

NLi: Writing – original draft. TW: Writing – original draft. HZ: Writing – original draft. XL: Writing – original draft. HB: Writing – original draft. NLu: Writing – original draft. KL: Writing – review & editing.

## References

[1] Bewersdorf JP Zeidan AM. Hyperleukocytosis and leukostasis in acute myeloid leukemia: can a better understanding of the underlying molecular pathophysiology lead to novel treatments? Cells. (2020) 9(10):2310. doi: 10.3390/cells9102310 33080779 PMC7603052

[2] Shah A Naqvi SS Naveed K Salem N Khan MA Alimgeer KS. Automated diagnosis of leukemia: a comprehensive review. IEEE Access. (2021) 9:132097–124. doi: 10.1109/ACCESS.2021.3114059

[3] Kuykendall A Duployez N Boissel N Lancet JE Welch JS. Acute myeloid leukemia: the good, the bad, and the ugly. Am Soc Clin Oncol Educ Book. (2018) 38:555–573. doi: 10.1200/EDBK_199519 30231330

[4] Grimwade D Lo Coco F. Acute promyelocytic leukemia: a model for the role of molecular diagnosis and residual disease monitoring in directing treatment approach in acute myeloid leukemia. Leukemia. (2002) 16:1959–73. doi: 10.1038/sj.leu.2402721 12357347

[5] Hallek M. Chronic lymphocytic leukemia: 2020 update on diagnosis, risk stratification and treatment. Am J Of Hematol. (2019) 94:1266–87. doi: 10.1002/ajh.25595 31364186

[6] Matés JM Segura JA Campos-Sandoval JA Lobo C Alonso L Alonso FJ. Glutamine homeostasis and mitochondrial dynamics. Int J Biochem Cell Biol. (2009) 41:2051–61. doi: 10.1016/j.biocel.2009.03.003 19703661

[7] Cruzat V Macedo Rogero M Noel Keane K Curi R Newsholme P. Glutamine: metabolism and immune function, supplementation and clinical translation. Nutrients. (2018) 10:1564. doi: 10.3390/nu10111564 30360490 PMC6266414

[8] Pavlova NN Thompson CB. The emerging hallmarks of cancer metabolism. Cell Metab. (2016) 23:27–47. doi: 10.1016/j.cmet.2015.12.006 26771115 PMC4715268

[9] Hensley CT Wasti AT DeBerardinis RJ. Glutamine and cancer: cell biology, physiology, and clinical opportunities. J Clin Invest. (2013) 123:3678–84. doi: 10.1172/JCI69600 PMC3754270 23999442

[10] Lukey MJ Wilson KF Cerione RA. Therapeutic strategies impacting cancer cell glutamine metabolism. Future medicinal Chem. (2013) 5:1685–700. doi: 10.4155/fmc.13.130 PMC4154374 24047273

[11] Sanderson E Glymour MM Holmes MV Kang H Morrison J Munafò MR. Mendelian randomization. Nat Rev Methods Primers. (2022) 2:6. doi: 10.1038/s43586-021-00092-5 37325194 PMC7614635

[12] Skrivankova VW Richmond RC Woolf BAR Davies NM Swanson SA VanderWeele TJ. Strengthening the reporting of observational studies in epidemiology using mendelian randomisation (STROBE-MR): explanation and elaboration. BMJ. (2021) 375:n2233. doi: 10.1136/bmj.n2233 PMC8546498 34702754

[13] Hemani G Zheng J Elsworth B Wade KH Haberland V Baird D. The MR-Base platform supports systematic causal inference across the human phenome. elife. (2018) 7:e34408. doi: 10.7554/eLife.34408 29846171 PMC5976434

[14] Taschler B Smith SM Nichols TE. Causal inference on neuroimaging data with Mendelian randomisation. NeuroImage. (2022) 258:119385. doi: 10.1016/j.neuroimage.2022.119385 35714886 PMC10933777

[15] Yarmolinsky J Wade KH Richmond RC Langdon RJ Bull CJ Tilling KM. Causal inference in cancer epidemiology: what is the role of Mendelian randomization? Cancer Epidemiology Biomarkers Prev. (2018) 27:995–1010. doi: 10.1007/978-3-319-42542-9_11 PMC6522350 29941659

[16] Marcelín-Jiménez G Angeles Moreno ACP Mendoza-Morales L Rivera-Espinosa L Martínez MM. Development of an ultra-performance liquid chromatography–tandem mass spectrometry micromethod for quantification of lamotrigine in human plasma and its use in a bioequivalence trial. Bioanalysis (2009) 1(1):47–55. doi: 10.4155/bio.09.8 21083187

[17] He S Bian J Shao Q Zhang Y Hao X Luo X. Therapeutic Drug Monitoring and Individualized Medicine of Dasatinib: Focus on Clinical Pharmacokinetics and Pharmacodynamics. Front Pharmacol. (2021) 12:797881. doi: 10.3389/fphar.2021.797881 PMC8685414 34938198

[18] Lucas AT Moody A Schorzman AN Zamboni WC. Importance and considerations of antibody engineering in antibody-drug conjugates development from a clinical pharmacologist’s perspective. Antibodies. (2021) 10:30. doi: 10.3390/antib10030030 34449544 PMC8395454

[19] Kably B Launay M Derobertmasure A Lefeuvre S Dannaoui E Billaud EM. trends and update. Ther Drug Monit. (2022) 44:166–97. doi: 10.1097/FTD.0000000000000952 34923544

[20] Siraj S. Therapeutic drug monitoring of selected drugs: an approach to drug therapy optimization, the tamilnadu Dr. Chennai: MGR Medical University (2012).

[21] Von Elm E Altman DG Egger M Pocock SJ Gøtzsche PC Vandenbroucke JP. The Strengthening the Reporting of Observational Studies in Epidemiology (STROBE) Statement: guidelines for reporting observational studies. Int J Surg. (2014) 12:1495–9. doi: 10.1016/j.ijsu.2014.07.013

[22] Skrivankova VW Richmond RC Woolf BA Yarmolinsky J Davies NM Swanson SA. Strengthening the reporting of observational studies in epidemiology using Mendelian randomization: the STROBE-MR statement. Jama. (2021) 326:1614–21. doi: 10.1001/jama.2021.18236 34698778

[23] Crivelari da Cunha M Gonçalves Dos Santos Aguilar J Orrillo Lindo SMDR Soares de Castro RJ Sato HH. L-asparaginase from Aspergillus oryzae spp.: Effects of production process and biochemical parameters. Preparative Biochem Biotechnol. (2022) 52:253–63. doi: 10.1080/10826068.2021.1931881 34110268

[24] Bonfiglio F Liu X Smillie C Pandit A Kurilshikov A Bacigalupe R. GWAS of stool frequency provides insights into gastrointestinal motility and irritable bowel syndrome. Cell Genom. (2021) 8(3). doi: 10.1016/j.xgen.2021.100069 PMC8654685 34957435

[25] Stroup DF Berlin JA Morton SC Olkin I Williamson GD Rennie D. Meta-analysis of observational studies in epidemiology: a proposal for reporting. Jama. (2000) 283:2008–12. doi: 10.1001/jama.283.15.2008 10789670

[26] Bowden J Del Greco MF Minelli C Davey Smith G Sheehan NA Thompson JR. Assessing the suitability of summary data for two-sample Mendelian randomization analyses using MR-Egger regression: the role of the I2 statistic. Int J Epidemiol. (2016) 45:1961–74. doi: 10.1093/ije/dyw220 PMC5446088 27616674

[27] Pagano M Gauvreau K Mattie H. Principles of biostatistics. Chapman Hall/CRC. (2022) 26–30. doi: 10.1201/9780429340512

[28] Jin T Huang W Pang Q He Z Yuan L Zhang H. Inferring the genetic effects of serum homocysteine and vitamin B levels on autism spectral disorder through Mendelian randomization. Eur J Nutr. (2024) 63(3):977–986. doi: 10.1007/s00394-024-03329-7 38265752

[29] Hao S Shen L Liu P Yong Q Wang Y Zheng X. Development of a prognostic model for muscle-invasive bladder cancer using glutamine metabolism. Comput Biol Med. (2024) 171:108223. doi: 10.1016/j.compbiomed.2024.108223 38430744

[30] Byun J-K Park M Lee S Yun JW Lee J Kim JS. Inhibition of glutamine utilization synergizes with immune checkpoint inhibitor to promote antitumor immunity. Mol Cell. (2020) 80:592–606.e8. doi: 10.1016/j.molcel.2020.10.015 33159855

[31] Wang B Pei J Xu S Liu J Yu J. A glutamine tug-of-war between cancer and immune cells: recent advances in unraveling the ongoing battle. J Exp Clin Cancer Res. (2024) 43:74. doi: 10.1186/s13046-024-02994-0 38459595 PMC10921613

[32] Yang L Achreja A Yeung T-L Mangala LS Jiang D Han C. Targeting stromal glutamine synthetase in tumors disrupts tumor microenvironment-regulated cancer cell growth. Cell Metab. (2016) 24:685–700. doi: 10.1016/j.cmet.2016.10.011 27829138 PMC7329194

[33] Yang L Venneti S Nagrath D. Glutaminolysis: a hallmark of cancer metabolism. Annu Rev Biomed Eng. (2017) 19:163–94. doi: 10.1146/annurev-bioeng-071516-044546 28301735

[34] Ruzza P Rosato A Rossi CR Floreani M Quintieri L. Glutathione transferases as targets for cancer therapy. Anti-Cancer Agents Medicinal Chem (Formerly Curr Medicinal Chemistry-Anti-Cancer Agents). (2009) 9:763–77. doi: 10.2174/187152009789056895 19538171

[35] Mohamed A Deng X Khuri FR Owonikoko TK. Altered glutamine metabolism and therapeutic opportunities for lung cancer. Clin Lung Cancer. (2014) 15:7–15. doi: 10.1016/j.cllc.2013.09.001 24377741 PMC3970234

[36] Guo C You Z Shi H Sun Y Du X Palacios G. SLC38A2 and glutamine signalling in cDC1s dictate anti-tumour immunity. Nature. (2023) 620:200–8. doi: 10.1038/s41586-023-06299-8 PMC10396969 37407815

[37] Cui JJ Wang LY Tan ZR Zhou HH Zhan X Yin JY. Mass spectrometry-based personalized drug therapy. Mass Spectrometry Rev. (2020) 39:523–52. doi: 10.1002/mas.21620 31904155

[38] Combs JA DeNicola GM. The non-essential amino acid cysteine becomes essential for tumor proliferation and survival. Cancers. (2019) 11:678. doi: 10.3390/cancers11050678 31100816 PMC6562400

[39] Hope HC Salmond RJ. The role of non-essential amino acids in T cell function and anti-tumour immunity. Archivum Immunologiae Therapiae Experimentalis. (2021) 69:29. doi: 10.1007/s00005-021-00633-6 PMC8510955 34637000

[40] Violi JP Bishop DP Padula MP Steele JR Rodgers KJ. Considerations for amino acid analysis by liquid chromatography-tandem mass spectrometry: A tutorial review. TrAC Trends Analytical Chem. (2020) 131:116018. doi: 10.1016/j.trac.2020.116018

[41] Manig F Kuhne K von Neubeck C Schwarzenbolz U Yu Z Kessler BM. The why and how of amino acid analytics in cancer diagnostics and therapy. J Biotechnol. (2017) 242:30–54. doi: 10.1016/j.jbiotec.2016.12.001 27932276

[42] Barbolosi D Ciccolini J Lacarelle B Barlési F André N. Computational oncology—mathematical modelling of drug regimens for precision medicine. Nat Rev Clin Oncol. (2016) 13:242–54. doi: 10.1038/nrclinonc.2015.204 26598946

[43] Tyson RJ Park CC Powell JR Patterson JH Weiner D Watkins PB. Precision dosing priority criteria: drug, disease, and patient population variables. Front Pharmacol. (2020) 11:420. doi: 10.3389/fphar.2020.00420 32390828 PMC7188913

[44] Chiu M Taurino G Bianchi MG Kilberg MS Bussolati O. Asparagine synthetase in cancer: beyond acute lymphoblastic leukemia. Front Oncol. (2020) 9:1480. doi: 10.3389/fonc.2019.01480 31998641 PMC6962308

[45] Van Trimpont M Peeters E De Visser Y Schalk AM Mondelaers V De Moerloose B. Novel insights on the use of L-asparaginase as an efficient and safe anti-cancer therapy. Cancers. (2022) 14:902. doi: 10.3390/cancers14040902 35205650 PMC8870365

[46] Schiliro C Firestein BL. Mechanisms of metabolic reprogramming in cancer cells supporting enhanced growth and proliferation. Cells. (2021) 10:1056. doi: 10.3390/cells10051056 33946927 PMC8146072

[47] Bott AJ Maimouni S Zong W-X. The pleiotropic effects of glutamine metabolism in cancer. Cancers. (2019) 11:770. doi: 10.3390/cancers11060770 31167399 PMC6627534

[48] Kuo MT Chen HH Feun LG Savaraj N. Targeting the proline–glutamine–asparagine–arginine metabolic axis in amino acid starvation cancer therapy. Pharmaceuticals. (2021) 14:72. doi: 10.3390/ph14010072 33477430 PMC7830038

[49] Ireland RJ Lea PJ. The enzymes of glutamine, glutamate, asparagine, and aspartate metabolism. New York: Marcel Dekker, Inc (1999).

[50] Reinert RB Oberle LM Wek SA Bunpo P Wang XP Mileva I. Role of glutamine depletion in directing tissue-specific nutrient stress responses to L-asparaginase. J Biol Chem. (2006) 281:31222–33. doi: 10.1074/jbc.M604511200 16931516

[51] Bhutia YD Ganapathy V. Glutamine transporters in mammalian cells and their functions in physiology and cancer. Biochim Biophys Acta (BBA)-Molecular Cell Res. (2016) 1863:2531–9. doi: 10.1016/j.bbamcr.2015.12.017 PMC4919214 26724577

[52] Yuan Q Yin L He J Zeng Q Liang Y Shen Y. Metabolism of asparagine in the physiological state and cancer. Cell Communication Signaling. (2024) 22:163. doi: 10.1186/s12964-024-01540-x 38448969 PMC10916255

[53] Avramis VI. Asparaginases: biochemical pharmacology and modes of drug resistance. Anticancer Res. (2012) 32(7):2423–37. 22753699

